# Food security status of *Suchana*-participating households in north-eastern rural Bangladesh

**DOI:** 10.3389/fpubh.2022.950676

**Published:** 2022-09-13

**Authors:** Md Ahshanul Haque, Nuzhat Choudhury, S. M. Tanvir Ahmed, Fahmida Dil Farzana, Mohammad Ali, Farina Naz, Ashfaque Khan, Barbie Zaman Wahid, Towfida Jahan Siddiqua, Rumana Akter, Sheikh Shahed Rahman, A. S. G. Faruque, Tahmeed Ahmed

**Affiliations:** ^1^Nutrition and Clinical Services Division, icddr, b, Dhaka, Bangladesh; ^2^Child Poverty Sector, Save the Children Bangladesh, Dhaka, Bangladesh; ^3^Johns Hopkins University, Dhaka, Bangladesh

**Keywords:** Food security, *Suchana* intervention, pre-post design, logistic regression, Bangladesh

## Abstract

Despite achieving remarkable progress, food insecurity remains a major public health challenge in Bangladesh, and severe food insecurity status has not been reduced in susceptible areas and vulnerable regions in Bangladesh. Wetlands that are susceptible to flooding can be found in Bangladesh's north-eastern Sylhet division. *Suchana*, a large-scale nutrition programme, implemented nutrition-specific and sensitive interventions in poor and very poor households in Sylhet and Moulvibazar districts in the north-eastern region of Bangladesh. The aim of this article is to assess the association between the *Suchana* intervention and household food security status among poor and very poor households in north-eastern rural Bangladesh using the *Suchana* baseline and endline survey databases. The baseline survey was conducted between November 2016 and February 2017, while the endline survey was undertaken 3 years later, during the same months. The outcome variable in this analysis was household food security status, which was measured using the Food and Nutrition Technical Assistance's Guideline. Descriptive statistics were used to summarize the data; after controlling for the union as a cluster and relevant covariates, a multiple multinomial logistic regression model was used to estimate the independent effect of the *Suchana* intervention as an exposure. Overall, 14.0% of households were food secure at the baseline survey (intervention: 14.1%, control: 14.0%) and 22.0% were food secure (intervention: 26.6%, control: 20.2%) at the endline survey. For households in the intervention area in comparison to the control area, the odds of being moderately food insecure [aOR: 1.36 (1.05, 1.76), *p* < 0.05], mildly food insecure [aOR: 1.83 (1.33, 2.51), *p* < 0.001], or food secure [aOR: 2.21 (1.47, 3.33), *p* < 0.001] compared to being severely food insecure was significantly higher. Thus, we infer that the 3 years of *Suchana* intervention marginally increased household food security status among the socio-economically disadvantaged population in north-eastern rural Bangladesh. If concerns regarding gender equity, women's education, and income-generating activities are addressed, the population could experience even greater benefits in food security. In order to overcome these challenges, all stakeholders including programme implementers and policymakers should work together to implement the appropriate measures.

## Introduction

The Sustainable Development Goals (SDGs) aim to address the most pressing issues related to human development through 15 goals that are sub-divided into measurable targets and indicators of progress. The first and second goals are dedicated to (1) ending poverty in all its forms everywhere, and (2) ending hunger, achieving food security and improved nutrition, and promoting sustainable agriculture (1). However, food insecurity has remained far above the levels needed to achieve these SDGs (2). By definition, household food insecurity is inadequate access to food that is sufficient, nutritionally safe, and meets the dietary needs to live an active and healthy life. Food insecurity causes hunger and malnutrition at the global level (3). In 2018, ~26.4% of the global population was affected by moderate or severe food insecurity (2). People suffering from moderate food insecurity are usually not able to eat a healthy, balanced diet on a daily basis due to a low income or other resource constraints (4).

Countries in Southern Asia are home to the second-largest poor and undernourished populations in the world and suffer extraordinary population health challenges, with the majority of their populations suffering extensive hunger (5). Food insecurity is a major public health challenge in Bangladesh, especially for women and children (6). In Bangladesh, mothers with children are at higher risk of food insecurity and inadequate food consumption due to economic barriers (7, 8) and there is a dose-response relationship between the degree of household food insecurity and several types of maternal and child health indicators, including malnutrition, dietary diversity, anemia, domestic violence, and healthcare practices (9–13). Bangladesh has made significant progress in reducing household food insecurity; nonetheless, the condition of acute food insecurity in Bangladesh's vulnerable regions—such as the coastal belt, eastern hills, *haor, padma chars*, and northern *char* region—is not improving.

Sylhet division in the north-eastern region of Bangladesh consists of the *haor* (wetland) and is often affected by flash floods. According to the findings of the DHS, in contrast to the overall scenario in Bangladesh where maternal and child malnutrition are decreasing, the status in the Sylhet division is worsening and there is significant inequality in this region with regard to the socio-economic profile of the households (14). Indicators of maternal and child healthcare practices are also lagging in this region, especially in the *haor* (wetland) region, where these indicators are very low. Household food insecurity status could be an important factor underlying the lack of improvement in health and healthcare practice indicators in this area.

*Suchana*, a large-scale nutrition programme, implemented nutrition-specific and -sensitive interventions for poor and very poor households in Sylhet and Moulvibazar districts under the Sylhet division, with the primary aim of reducing the rate of stunting among children. One of the secondary aims of this programme in terms of improving and diversifying household income was to reduce food insecurity, which is a household nutritional and social behavioral indicator (15).

*Suchana* focused on strengthening household livelihood strategies so that households can afford a nutritious diet, have the financial resources to incorporate optimal nutrition practices, and invest in their future. Women and adolescent girls played a key part in the programme's market-driven approach, which provided investment grants or access to voluntary savings and loan associations (VSLAs) to invest in income-generating activities (IGAs). At the same time, broader market strengthening measures facilitated pro-poor market conditions, to ensure the long-term sustainability of the livelihood strategies after the programme ended. The goals of the intervention were to (i) encourage the production of nutritious food at home, primarily for personal consumption, as well as activities that protect against sudden and seasonal climatic shocks and (ii)reconnect producers with suppliers of goods and services, as well as other market actors, in order to ensure long-term sustainability. The aim of *Suchana* was to achieve a significant reduction (50%) in the proportion of food insecure households, according to the Household Food Insecurity Access Scale (HFIAS). The aims of this article are to investigate the factors associated with household food security status and to assess the association between the *Suchana* intervention and household food security status among poor and very poor households in Sylhet division in the north-eastern region of rural Bangladesh.

## Methods

### Study design and population

*Suchana*, a large-scale development programme, was implemented and enrolled its study population from poor and very poor households in vulnerable villages in Sylhet division in the north-eastern of Bangladesh. Using a range of nutrition-specific and nutrition-sensitive interventions, the *Suchana* programme addressed a total of 2,35,579 poor and extremely poor beneficiary households (BHHs) across 157 unions in 20 sub-districts. Unions were assigned in four phases at random. All four phases received the full set of interventions at the same time. The interventions in the *Suchana* programme were carried out for 36 months in each group (15). The phases of the *Suchana* programme are depicted in Supplementary Figure 1, along with the target BHHs and programme timeline. The vulnerable villages in each union were chosen by the programme staff based on their vulnerability (e.g., poverty or household living conditions, geographic isolation or difficulty in reaching the household, other development initiatives providing low or no interventions, flooding or submerging, and prevailing superstitions or strong social stigmas). This selection process was conducted after meetings and discussions with elected councils, local leaders, local government officials, and field visits (16). Once the villages were identified, wealth-ranking sessions were completed in each village. The most vulnerable households were identified, listed, and verified following the *Suchana* programme inclusion criteria and, if eligible for the study, given an identification number. The criteria for inclusion and exclusion are given in Supplementary Table 1.

A pre-post design was used for evaluation; Phase-1 and Phase-4 were selected for the evaluation. Phase-1 was considered an intervention group and Phase-4 was considered the control group. Two cross-sectional studies (baseline and endline) were employed among beneficiary women with at least one child aged <24 months from randomly selected vulnerable households. The baseline survey was undertaken between November 2016 and February 2017 before the intervention began, and the endline survey was conducted between November 2019 and February 2020 after the intervention had ended in Phase-1. For the purpose of data collection, the evaluation team sampled eight villages from each union at the baseline survey and 12 villages at the endline survey. Then, a sampling frame was prepared based on the wealth ranking and verified household list. The sample sizes were estimated based on *Suchana*'s primary outcome of childhood stunting and secondary outcomes of children exclusively breastfed and the minimum acceptable diet (15). Data from 5,440 households from the baseline survey and 10,722 households from the endline survey were included in this analysis (16).

### Outcome variable

The outcome variable in this paper was household food security status. The four dimensions of food and nutrition security are availability, accessibility, use and utilization, and stability. The accessibility dimension was used to measure the degree of household food insecurity in this study (17). Access is ensured when all households have enough resources to obtain food in sufficient quantity, quality, and diversity for a nutritious diet. Access to food mainly depends on the number of household resources and the price of the products consumed. The Household Food Insecurity Access Scale (HFIAS) was employed to quantify food insecurity following the Food and Nutrition Technical Assistance's Guideline (18), which is a continuous measure of the degree of food insecurity in a household. To evaluate the level of anxiety and uncertainty of household members about household food supply and assess inappropriate quality of food and inadequate food intake, we employed a questionnaire containing nine questions related to whether household members are worried about whether they would not have sufficient food and whether they would be unable to eat the kinds of foods they prefer due to a lack of resources, have to eat only a few kinds of foods due to an absence of resources, have to eat foods that they actually do not want to eat due to a shortage of resources, have to eat smaller meal portions than they felt they needed due to an absence of sufficient food, have to eat fewer meals in a day because there was insufficient food, if there was ever no food of any kind to eat in their household due to a lack of resources to purchase food, if they ever went to bed at night hungry because there was an insufficient amount of food, and if they ever went to bed at night without eating anything because sufficient food was not present/was lacking. Household food insecurity status was categorized as food secure, mildly food insecure, moderately food insecure, or severely food insecure (9, 18).

### Covariates

A list of several covariates was finalized through descriptive analysis, as well as a literature review.

#### Households' socio-demographic characteristics

Information on religion, level of education of the head of the household, occupation of the head of the household, and household size were used as household demographic characteristics. Ownership of household assets, floor material, main roof material, external wall material, number of dwelling rooms, type of latrine, and sources of drinking water was assessed as key indicators of socioeconomic status (SES). Using this information, factor analysis based on the principal factor method was used to create an asset index for SES (16).

#### Women's general characteristics

Several maternal characteristics were included as covariates in this study and adjusted for in the multiple models to assess the independent effect of the intervention on household food security. The maternal indicators were current age, the number of children, visits by NGO health professionals, the experience of any domestic violence, decision-making power, level of education, minimum dietary diversity, and income-generating activities (9).

#### Other covariates

Covariates such as selected children (for the *Suchana* intervention) experiencing any illness in the last 15 days; selected children were stunted, household members involved in or receiving loans, incidents occurring in the last year, households with access to cultivable land, per capita household income, household membership of a co-operative/savings committee, household involvement with horticulture and poultry farms, and household receipt of any grant/allowance/stipend from the government were also analyzed as covariates in this article. The per capita income was defined as household income/(family size)∧0.56 (19). The variable was treated as a binary variable, indicating (1) if per capita yearly income was equal to or above the median (>33,126 BDT) and (0) if per capita income was below the median.

The variable “incidents occurring in the last year” was defined as any crisis or adverse events experienced by the households in the last 1-year period, such as severe damage to the house, severe illness of earning member(s), severe illness of a non-earning member(s), crop loss due to natural disaster, death of earning member(s), the unnecessary cost in marriage(s) of household members, loss of livestock, legal dispute(s), and theft from the household.

### Statistical analysis

### Data management

The *Suchana* data collection software had in-built validation rules. Maximum validation rules were set in the data system to prevent errors during data entry, such as uniqueness, requirements, skipping rules, value ranges, conditional fields, and the number of digits that could be entered. During the data entry period, activities such as editing (after receiving any feedback from field staff members), updates, range checks, duplication checks, consistency checks, frequency checks, and cross-tabulation were performed regularly. In the case of any unusual observations, the issues were discussed and resolved (16).

### Descriptive statistics

We used STATA to analyze the data upon entry. Several statistical plots, such as histograms, bar diagrams, pie charts, and scatterplots, were used for data visualization. Descriptive statistics were used to summarize the data, such as frequencies and proportions for categorical variables, mean and SD values for symmetric quantitative variables, and median and interquartile ranges (IQR) for asymmetric quantitative variables. The outcome variables and all covariates were segregated by the baseline vs. endline survey and the intervention vs. control group.

### Explorative statistics

To test the hypothesis of interest that *Suchana* achieved a significant improvement in the prevalence of household food security status, simple multinomial logistic regression analysis was primarily used to explore the bivariate association between the outcome variable and the intervention variable (using the *Suchana* control group as the reference category). To estimate the independent effect of the *Suchana* intervention as an exposure, a multiple multinomial logistic regression model was employed after controlling for relevant covariates. Covariates with a bivariate relationship in the multinomial logistic regression analysis, as well as covariates identified in a literature review, were included in the multiple models. Union was adjusted as a cluster variable. In all analyses, *p* < 0.05 were considered significant.

## Results

### General characteristics

A total of 16,158 women were interviewed: 5,440 (intervention: 2,720; control: 2,720) in the baseline study and 10,722 (intervention: 5,282; control: 5,440) in the endline study. The socio-demographic characteristics of the households and the women's general characteristics are presented in Table 1.

**Table 1:** Household and women's general characteristics.

**Indicators, % (*n*)**	**Baseline**		**Endline**	
	**Intervention**	**Control**	**Intervention**	**Control**
**Household characteristics**				
Male household head	95.99 (2,611)	96.80 (2,633)	92.41 (4,880)	92.48 (5,028)
Age of household head^*^	39.30 ± 13.00	40.20 ± 13.20	38.50 ± 10.50	38.60 ± 12.10
Household head education: no “schooling”	49.02 (1,333)	48.86 (1,329)	44.26 (2,337)	40.68 (2,212)
Household size^*^	6.12 ± 2.31	6.48 ± 2.56	6.13 ±2.11	5.94 ± 2.27
Religion: *Muslim*	89.62 (2,438)	92.86 (2,526)	91.67 (4,841)	92.44 (5,026)
Household dietary diversity score ≥ 7	65.96 (1,794)	69.08 (1,879)	88.90 (4,694)	78.35 (4,260)
Median household income/year (BDT)	80,000	83,012	96,000	86,000
Per capita yearly income ≥ 33,126 BDT	40.65 (1,083)	45.11 (1,209)	55.44 (2,807)	50.42 (2,606)
**Asset index**				
1st quintile	20.26 (551)	19.74 (537)	18.73 (989)	21.24 (1,155)
2nd quintile	20.92 (569)	19.08 (519)	19.96 (1,054)	20.08 (1,092)
3rd quintile	19.71 (536)	20.29 (552)	20.58 (1,087)	19.39 (1,054)
4th quintile	20.77 (565)	19.23 (523)	20.98 (1,108)	19.05 (1,036)
5th quintile	18.35 (499)	21.65 (589)	19.75 (1,043)	20.23 (1,100)
Household involved with any loan	71.91 (1,956)	71.43 (1,943)	79.47 (4,197)	74.77 (4,065)
Household has access to cultivable land	30.29 (824)	30.99 (843)	43.39 (2,292)	40.81 (2,220)
Household membership of co-operative/ savings committee	33.30 (905)	32.00 (869)	50.75 (2,680)	33.92 (1,844)
Household received any grant/allowance/stipend from the government	22.06 (600)	21.43 (583)	27.81 (1,468)	24.84 (1,348)
Incidents occurring in the last year	69.30 (1,885)	70.63 (1,921)	69.81 (3,684)	68.36 (3,712)
**Women's general characteristics**				
Current age^*^	26.87 ± 5.61	26.92 ± 5.67	29.15 ± 5.31	27.27 ± 5.71
Age at first pregnancy^*^	19.24 ± 2.87	19.40 ± 2.94	19.73 ± 3.12	19.79 ± 3.21
Age at marriage^*^	18.14 ± 2.65	18.34 ± 2.77	18.68 ± 2.94	18.88 ± 3.08
**Number of children**				
1	21.03 (572)	21.73 (591)	3.52 (186)	21.39 (1,163)
2–3	44.23 (1,203)	41.36 (1,125)	51.73 (2,732)	46.88 (2,549)
4+	34.74 (945)	36.91 (1,004)	44.75 (2,363)	31.73 (1,725)
**Education**				
No schooling	22.32 (607)	23.75 (646)	17.91 (946)	14.66 (797)
Primary incomplete	22.72 (618)	21.14 (575)	23.37 (1,234)	22.51 (1,224)
Primary complete	54.96 (1,495)	55.11 (1,499)	58.72 (3,101)	62.83 (3,416)
Not involved in any earning activities	97.06 (2,640)	97.10 (2,641)	87.29 (4,610)	93.80 (5,100)
Does not get any support from household members	5.44 (148)	5.77 (157)	3.43 (181)	4.10 (223)
Minimum dietary diversity for women (at least five groups)	25.92 (705)	27.46 (747)	52.58 (2,776)	33.53 (1,824)
Visits from NGO health professionals	27.50 (748)	17.10 (465)	39.92 (2,108)	20.05 (1,090)
**Domestic violence and abuse**				
Husband threatening divorce	7.46 (203)	6.80 (185)	9.35 (494)	11.44 (622)
Husband threatening to take another wife	7.87 (214)	6.99 (190)	10.68 (564)	12.31 (669)
Verbal abuse by husband/other family member(s)	33.79 (919)	31.32 (852)	43.14 (2,278)	41.92 (2,279)
Physical abuse by husband/other family member(s)	13.75 (374)	13.38 (364)	17.97 (949)	19.32 (1,050)
Experienced any domestic violence	36.07 (981)	33.27 (905)	44.57 (2,354)	43.65 (2,373)
**Women have decision-making power on**				
Food purchase	44.56 (1,212)	43.42 (1,181)	74.66 (3,943)	63.95 (3,477)
Major household purchase	25.22 (686)	24.34 (662)	55.77 (2,945)	41.14 (2,237)
Food preparation	78.13 (2,125)	75.77 (2,061)	87.03 (4,596)	80.38 (4,370)
Child health care	51.25 (1,394)	50.63 (1,377)	76.96 (4,064)	67.56 (3,673)
Own health care	58.86 (1,601)	56.32 (1,532)	79.93 (4,221)	71.07 (3,864)
Visiting family and relatives	42.65 (1,160)	42.90 (1,167)	66.50 (3,512)	55.67 (3,027)
All types of decision making	17.32 (471)	16.80 (457)	45.26 (2,390)	31.38 (1,706)
Selected children experienced any illness in the last 15 days	44.41 (1,208)	43.68 (1,188)	42.20 (2,294)	41.54 (2,193)
Selected children were stunted	49.60 (1,349)	50.99 (1,387)	44.08 (2,398)	45.13 (2,383)

* Mean ± SD.

**Figure 1:**
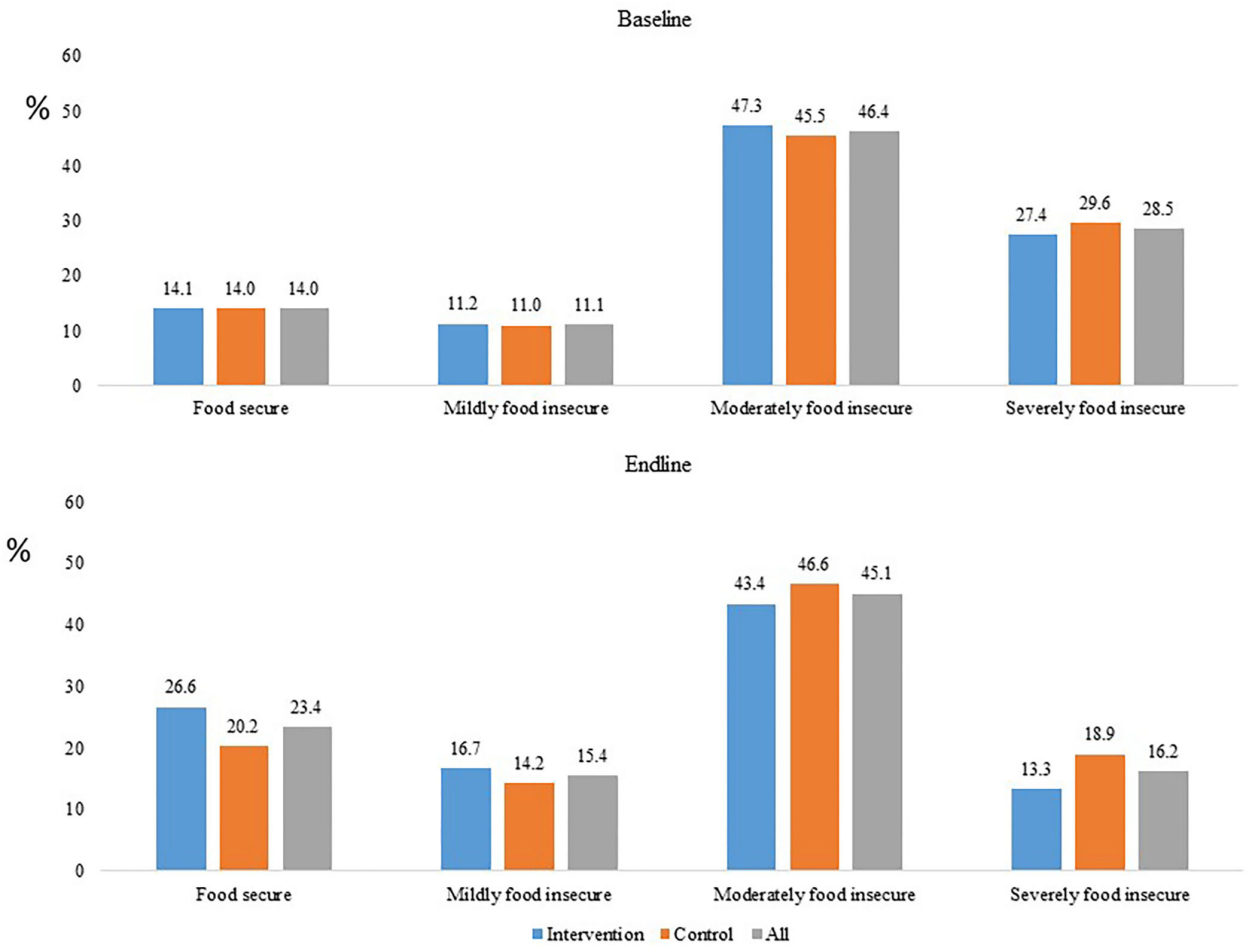
Household food security status segregated by the baseline vs. endline surveys and intervention vs. control groups.

At baseline, 14.0% of households were food secure overall (intervention: 14.1%, control: 14.0%). The remaining households suffered some degree of food insecurity, with 11.1% mildly food insecure (intervention: 11.2%, control: 11.0%), 46.4% moderately food insecure (intervention: 47.3%, control: 45.5%), and 28.5% severely food insecure (intervention: 27.4%, control: 29.6%). However, at endline, 23.4% of households were food secure (intervention: 26.6%, control: 20.2%), 15.4% were mildly food insecure (intervention: 16.7%, control: 14.2%), 45.1% were moderately food insecure (intervention: 43.4%, control: 46.6%), and 16.2% were severely food insecure (intervention: 13.3%, control: 18.9%; Figure 1).

### Factors affecting food security

The adjusted odds ratios calculated using multiple multinomial logistic regression analyses of the associations between various factors and food security status are presented in Table 2. The adjusted analysis showed that the education and sex of the household head, household size, household asset index, household loan status, household access to cultivable land, household membership of a co-operative/savings committee, household involvement with horticulture and poultry farming, incidents occurring in the last year, household per capita income, respondent's current age, respondent's number of children, the maternal experience of any domestic violence, maternal decision-making power, mother receiving any support from household members, maternal visits from NGO health professionals, the selected child experienced any illness in the last 15 days, and the nutritional status of the selected child was associated with household food security.

**Table 2:** Factors associated with household food security status.

**Household food security status**	**Food secure**		**Mildly food insecure**		**Moderately food insecure**	
	**Adjusted mOR (95% CI)**	***P*** **-value**	**Adjusted mOR (95% CI)**	***P*** **-value**	**Adjusted mOR (95% CI)**	***P*** **-value**
	**Severely food insecurity is the base outcome in the multinomial model**					
**Educational level of household head**				
No schooling	Reference		Reference		Reference	
At least 1 year of formal education	1.43 (1.23, 1.67)	0.000	1.30 (1.14, 1.48)	0.000	1.14 (1.03, 1.27)	0.015
**Sex of household head**				
Male	Reference		Reference		Reference	
Female	0.97 (0.75, 1.24)	0.794	0.68 (0.48, 0.96)	0.028	0.73 (0.58, 0.93)	0.011
**Age of household head**	1.00 (0.99, 1.00)	0.631	1.00 (0.99, 1.00)	0.276	1.00 (1.00, 1.01)	0.368
**Household size**	0.92 (0.89, 0.95)	0.000	0.90 (0.87, 0.94)	0.000	0.95 (0.93, 0.97)	0.000
**Asset index**				
1st quintile	Reference		Reference		Reference	
2nd quintile	1.71 (1.35, 2.16)	0.000	1.62 (1.33, 1.98)	0.000	1.53 (1.34, 1.75)	0.000
3rd quintile	2.50 (1.99, 3.14)	0.000	2.41 (1.98, 2.94)	0.000	1.90 (1.63, 2.22)	0.000
4th quintile	4.02 (2.92, 5.54)	0.000	3.70 (2.95, 4.65)	0.000	2.49 (2.07, 3.00)	0.000
5th quintile	9.85 (7.1, 13.68)	0.000	6.64 (5.05, 8.74)	0.000	3.30 (2.65, 4.11)	0.000
**Household has any loan**				
Yes	Reference		Reference		Reference	
No	1.99 (1.68, 2.36)	0.000	1.34 (1.15, 1.58)	0.000	1.01 (0.90, 1.14)	0.892
**Household has access to cultivable land**				
No	Reference		Reference		Reference	
Yes	1.89 (1.62, 2.20)	0.000	1.84 (1.63, 2.07)	0.000	1.42 (1.27, 1.60)	0.000
**Membership of co-operative/savings committee**				
No	Reference		Reference		Reference	
Yes	1.49 (1.27, 1.76)	0.000	1.41 (1.20, 1.65)	0.000	1.16 (1.04, 1.30)	0.011
**Household involved in horticulture**				
No	Reference		Reference		Reference	
Yes	1.44 (1.21, 1.71)	0.000	1.48 (1.25, 1.75)	0.000	1.36 (1.20, 1.54)	0.000
**Household involved in poultry farming**				
No	Reference		Reference		Reference	
Yes	1.27 (1.02, 1.57)	0.030	1.22 (0.98, 1.51)	0.070	1.25 (1.09, 1.43)	0.002
**Per capita income above median**				
No	Reference		Reference		Reference	
Yes	4.37 (3.71, 5.14)	0.000	2.76 (2.37, 3.23)	0.000	1.74 (1.56, 1.95)	0.000
**Incidents occurred in last year**				
Yes	Reference		Reference		Reference	
No	2.71 (2.25, 3.26)	0.000	2.35 (2.04, 2.71)	0.000	1.60 (1.41, 1.81)	0.000
**Religion**				
Muslim	Reference		Reference		Reference	
Non-Muslim	1.20 (0.95, 1.50)	0.119	1.18 (0.91, 1.52)	0.207	1.08 (0.88, 1.33)	0.452
**Respondent's current age**	0.98 (0.97, 0.99)	0.005	0.99 (0.98, 1.00)	0.201	0.99 (0.98, 0.99)	0.001
**Respondent's number of children**				
More than one	Reference		Reference		Reference	
One	1.19 (1.00, 1.42)	0.055	1.13 (0.95, 1.35)	0.172	1.09 (0.96, 1.23)	0.172
**Respondent's experience of any domestic violence**				
Yes	Reference		Reference		Reference	
No	1.89 (1.62, 2.21)	0.000	1.19 (1.01, 1.39)	0.037	1.08 (0.98, 1.21)	0.132
**Respondent has decision making power**				
No	Reference		Reference		Reference	
Yes	1.21 (1.02, 1.43)	0.025	1.09 (0.92, 1.28)	0.302	1.10 (0.98, 1.25)	0.116
**Respondent receives any support from household members**				
No	Reference		Reference		Reference	
Yes	1.56 (1.15, 2.12)	0.005	2.21 (1.44, 3.38)	0.000	1.29 (0.98, 1.69)	0.071
**Respondent's visits from NGO health professionals**				
No	Reference		Reference		Reference	
Yes	1.10 (0.89, 1.36)	0.372	1.08 (0.89, 1.31)	0.430	1.14 (0.99, 1.31)	0.063
**Selected child experienced any illness in the last 15 days**				
Yes	Reference		Reference		Reference	
No	1.32 (1.19, 1.46)	0.000	1.29 (1.14, 1.47)	0.000	1.08 (0.98, 1.19)	0.108
**Selected child was stunted**				
Yes	Reference		Reference		Reference	
No	1.21 (1.08, 1.34)	0.001	1.10 (0.98, 1.25)	0.106	1.05 (0.96, 1.16)	0.289

Union was used as a cluster in the multinomial logit model, both baseline and endline data were assessed in this analysis.

### Association of the *Suchana* intervention with household food security status

Based on the HFIAS, the odds of being moderately food insecure [aOR: 1.36 (1.05, 1.76), *p* < 0.05], mildly food insecure [aOR: 1.83 (1.33, 2.51), *p* < 0.001], or food secure [aOR: 2.21 (1.47, 3.33), *p* < 0.001] rather than severely food insecure were significantly higher for households in the intervention group compared to the control group (Table 3).

**Table 3:** Association between the *Suchana* intervention and household food security status.

	**Baseline survey**		**Endline survey**	
**Household food security status**	**Adjusted mOR (95% CI)**	***P*** **-value**	**Adjusted mOR (95% CI)**	***P*** **-value**
Severely food insecure	Base outcome		Base outcome	
Moderately food insecure	1.10 (0.90, 1.33)	0.354	1.36 (1.05, 1.76)	0.019
Mildly food insecure	1.07 (0.74, 1.53)	0.732	1.83 (1.33, 2.51)	<0.001
Food secure	1.14 (0.72, 1.80)	0.583	2.21 (1.47, 3.33)	<0.001

Adjusted in multinomial logit model for sex of household head, age of household head, household head education, household size, household asset index, the household has any loan, the household has access to cultivable land, household membership of a co-operative/savings committee, incidents occurred in last year, religion, respondent's experience of any domestic violence, respondent's current age, respondent's number of children, respondent's decision making power, the respondent receives any support from household members, respondent received visits from NGO health professionals, the selected child experienced any illness in the last 15 days, and the selected child was stunted. Union was used as a cluster. The *Suchana* intervention was assessed as an exposure variable; the control group was treated as the reference group in the model.

## Discussion

This study explored the association between the *Suchana* intervention and household food security status among poor and very poor households in a vulnerable region of rural Bangladesh. At baseline, around one-seventh of the households were food secure and one-fourth of the households were severely food insecure, whereas the prevalence of food security significantly increased to one-fourth of households at the endline. *Suchana* aimed to achieve a household food security prevalence of 50%, yet only half of this target value was achieved. The national Food Security and Nutritional Surveillance Project (FSNSP) in 2014–16 (20) indicated that one-fifth of households in the wetland region of Sylhet division were severely food insecure, which is similar to the value obtained in the baseline survey. However, the odds ratio of households being severely food insecure in the Sylhet division decreased by seven-fold after the intervention. Another study conducted in the same region immediately after the flash floods of 2107 found that 38% of households overall and 24% of ultra-poor households were food-secure (21); these values are higher than our survey data. However, *Suchana* only enrolled poor and very poor households in dire financial distress from the most vulnerable villages (15). Therefore, *Suchana* targeted households with very low food security status. Our multinomial analysis revealed that the odds of having food security status (compared to severe food insecurity) increased by 2.2-fold in the intervention group. Although this achievement was statistically significant, the increase in the percentage of food secure households was below the expected target.

In our multivariable model of associated factors, we found that several households, respondents, and children's characteristics were significantly associated with household food security. SES, as assessed using the household asset index, has major implications for food security as the strength of the association between food security and severe food insecurity was relatively strong for the 5th quintile compared to the lowest quintile of SES status, which indicates that SES may improve if family food insecurity is decreased. The status of other economic and livelihood indicators, such as household membership of a co-operative/savings committee, per capita income, household members engaged in horticulture and poultry, respondents engaged in income-generating activities, and household access to cultivable land, did not significantly improve in the study population. Bangladesh is an agricultural country, and there was formerly a strong link between access to agricultural land and increased food security. Previous research found that economic status, as well as access to arable land, are both important factors that affect food security (22–24). According to the FAO, livestock is a major source of income for farmers in developing countries and contributes to food security. Money can be made by selling livestock products to ensure food security (24, 25). Additionally, a significant proportion of the households in our study population received loans, and the occurrence of unfavorable incidents was also high; both of these factors contributed to a greater level of food insecurity. The intervention also encouraged residents of this flood-affected area to engage in income-generating activities, including farming. The intervention also familiarized the participants with several NGOs. Because of this, it became possible for individuals to obtain loans from NGOs so that they could begin farming and other income-generating activities. Moreover, education did not improve and is another crucial element that helps to lower the prevalence of food insecurity (26).

Other indicators related to the respondents, such as the experience of any domestic violence, decision-making power, getting any support from household members, and visits from NGO health professionals, were also associated with the food security of the household. In Africa, ownership of poultry by rural families reduced poverty, improved food security, and promoted gender equality—especially among unprivileged households in rural areas (24, 27). However, we assessed the associations by calculating odds ratios in multinomial logistic regression. Thus, these associations could be symmetric relationships, since many studies have shown that food insecurity status is one predictor of domestic violence and empowerment (9, 28, 29). However, improving these indicators might enhance food security status. Morbidity and childhood stunting were also identified as important factors that influence household food insecurity (30). Our data revealed that food security was also associated with the children's having any illness in the previous 15 days and stunting of the target children.

After adjusting for these indicators in the multivariable model, we found that the *Suchana* intervention had a significant impact on household food security. However, we believe that it is critical to take additional steps to improve food security, such as expanding access to agricultural land, poultry, and other livestock-rearing activities, reducing the burden of household loans, reducing domestic violence, empowering women to make decisions on a variety of household issues, and providing assistance with household chores, in order to improve women's socioeconomic status so they can participate in more income-generating activities. Several types of crises or adverse events occur in the respondents' daily lives. Some of these events occur spontaneously, while others are the result of social obligations. In such circumstances, arrangements must be made by policy makers or at the government level to ensure that the affected families are not financially disadvantaged.

It is important to highlight that our data collection concluded in February 2020 and the COVID-19 pandemic was declared just a few months later, in March 2020. According to the literature, the pandemic has significantly impacted food security in Bangladesh's rural areas (31). The COVID-19 pandemic may further deteriorate the food security status—and eventually the nutritional status—of *Suchana* beneficiaries. However, to maintain the improvements achieved during the programme and prevent further deterioration, the population of this region may need extra support with some specific indicators. The *Suchana* intervention increased food security. However, if no further intervention is given to these poor and very poor households, these destitute people are likely to become nutritionally deprived.

Appropriate financial interventions and varied types of agricultural training sessions could potentially achieve increases in indicators such as households' access to cultivable land, membership of a co-operative/savings committee, involvement in horticulture, involvement in poultry farming, and per capita income, and turn, these changes could improve food security. To protect against the loss of crops due to natural disasters, household members could use the advanced climate resilience production technologies associated with agricultural activities that were included in the *Suchana* intervention. Improving the earning capacity of the poor and vulnerable sectors of the population, as well as the cost-effective implementation of targeted food programmes, are required to boost poor people's access to food. Hence, the government can implement a variety of strategic initiatives in order to identify the basic requirements of Bangladesh's most vulnerable population in the north-eastern region, such as food security. First, policies to ensure food security and access to food could be addressed in Bangladesh. Similarly, policies could be incorporated to improve farming systems and livelihoods through coordinating public and private organizations and implementing appropriate programmes to benefit rural communities, farmer groups, and households. Agriculture, water, education, transportation, health, and other public sector agencies could be strengthened to enable better design and management of food security programmes. Additionally, to increase agricultural production and lessen detrimental environmental effects, food security and sustainable agriculture initiatives could be integrated with currently implemented national programs. Furthermore, to ensure food self-sufficiency, a transparent regulatory environment that encourages private investment and boosts farm productivity could be established. Effective programmes and policies could be developed to provide the required support to communities and sectors exposed to natural disasters, the consequences of climate change, and food insecurity. To improve domestically based agricultural employment and productivity, regional agricultural trade and procurement strategies can be devised. These strategies could also include support for the agro and dairy industries. Public awareness of the necessity of food and nutrition security for a healthy living could be increased, as price regulation is crucial to increase the purchasing power of people with low incomes. At the system and family levels, women have a key role in the alleviation of food insecurity. Women in Bangladesh experience significant difficulties as a result of social and cultural conventions that limit their ability to fully engage in the economy, and they must overcome extreme obstacles to fully realize economic gains. Limited mobility, domestic violence, lack of visits from NGO health professionals, lack of opportunities for leadership positions, and lack of access to finance, market knowledge, agricultural supplies, or extension services are all examples of such hurdles. Several obstacles must be overcome, and counseling interventions may help to implement and achieve the empowerment of women in order to reduce food insecurity.

## Strength and limitation

The key strength of this article is its cluster randomized pre-post trial design, which provided strong evidence of the effects of the outcome indicators. Similar findings for the control and intervention groups at baseline indicate the homogeneity of the background characteristics and all indicators between the two study groups. Furthermore, the large sample size, use of appropriate techniques for selecting poor and very poor households, and proper methodology for sampling and statistical analyses are also strengths of this study. A possibility of recall bias remains regarding the HFIAS data, as information for the month preceding the survey was gathered through maternal responses. However, the large sample size and adjustment for relevant covariates in the regression model helps to minimize the bias. Since one of the inclusion criteria of this study was children aged 0–23 months, the data collectors faced high dropout rates due to the time gap between verification/screening and the time of data collection from the targeted households. When required, we replaced any household in the sampling frame by selecting the immediately previous household in an anti-clockwise direction, in order to survey the required number of households by phase and by age group according to our randomly generated listing.

## Conclusion

Overall, the 3 years of *Suchana* intervention marginally increased household food security status among the socioeconomically disadvantaged population in north-eastern rural Bangladesh. Furthermore, household food security status was positively associated with the household head's education level, participation in income-generating activities, access to cultivable land, membership in co-operatives/saving committees, participation in horticulture/poultry, increased asset ownership, reducing exposure to events, reliance on loans, and experience with domestic violence. These findings indicate that further improvements to these indicators have the potential to improve household food security status in a similar population. To achieve this, support from the government of Bangladesh as well as from non-government organizations and concerned stakeholders can play a pivotal role.

## Data availability statement

The raw data supporting the conclusions of this article will be made available by the authors, without undue reservation.

## Ethics statement

This study was approved by the Research Review Committee and Ethical Review Committee, the two obligatory components of the Institutional Review Board (IRB) of icddr,b. Written informed consent to participate in this study was provided by the participants' legal guardian/next of kin.

## Author contributions

TA and NC originated the idea for the study and led the protocol design. MH conceptualized the manuscript. SA, SR, MH, NC, FF, and TA contributed to the survey design. MH performed the statistical analysis and drafted the manuscript. NC and AF supervised the work, critically reviewed, and provided feedback for revising the manuscript. MH, NC, MA, FF, FN, AK, RA, BW, TS, AF, TA, and SR contributed to the revision of the final draft for submission. All authors are responsible for the final content of the manuscript.

## Funding

This study was made possible by the committed contribution of the Foreign, Commonwealth and Development Office (FCDO) and icddr,b.

## Conflict of interest

The authors declare that the research was conducted in the absence of any commercial or financial relationships that could be construed as a potential conflict of interest.

## Publisher's note

All claims expressed in this article are solely those of the authors and do not necessarily represent those of their affiliated organizations, or those of the publisher, the editors and the reviewers. Any product that may be evaluated in this article, or claim that may be made by its manufacturer, is not guaranteed or endorsed by the publisher.
