# Supplementary material for: Food security status of Suchana-participating households in north-eastern rural Bangladesh
Source: Front Public Health. 2022 Sep 13;10:950676. doi: 10.3389/fpubh.2022.950676 (PMC9513544; doi:10.3389/fpubh.2022.950676)
Supplement: Supplementary file 1 [file Data_Sheet_1.PDF]

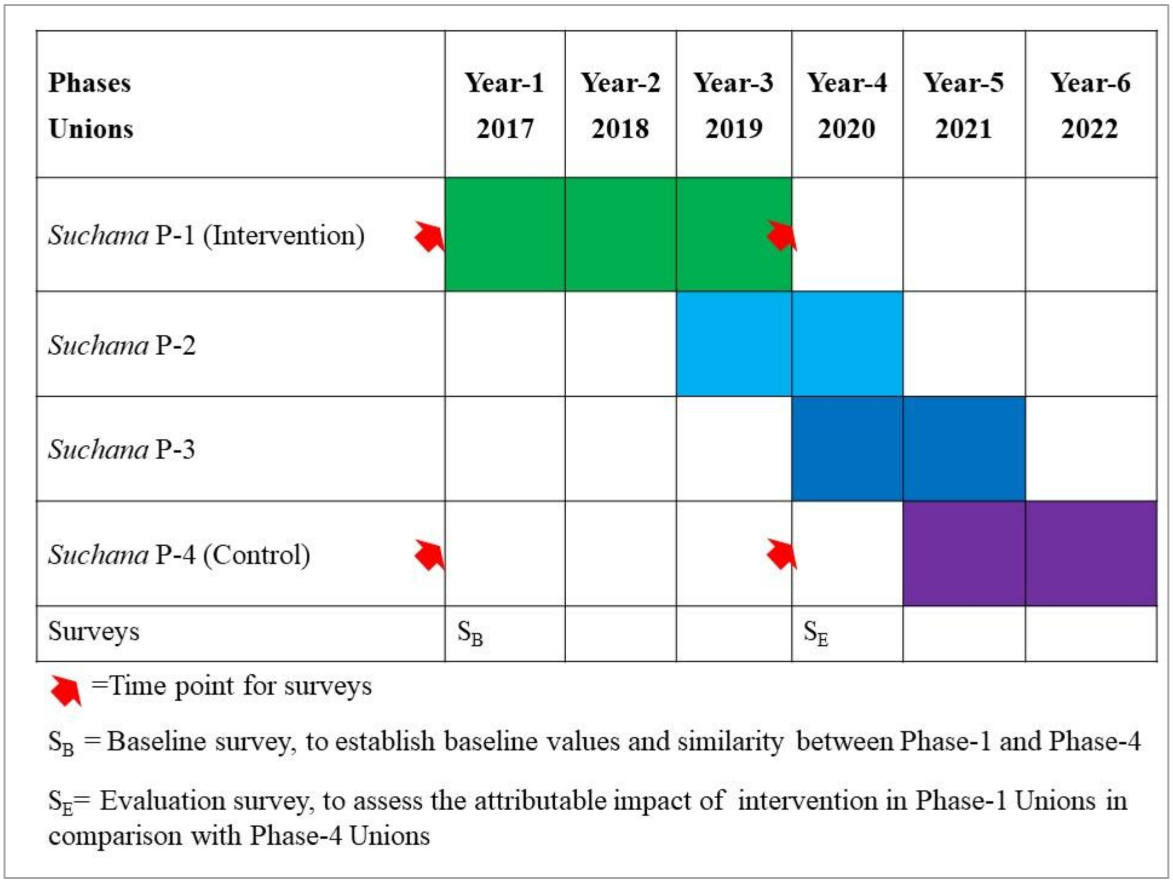

Supplementary Figure 1. The evaluation diagram of *Suchana* programme

Supplementary Table 1. *Suchana* inclusion criteria for registration of enrolling as vulnerable households

Vulnerable household verification questions	Inclusion criteria
Step 1 • Households currently participating/member of any livelihood, food security or asset transfer program	If "NO" go ahead for next questions
Step 2 • Ability to afford three (3) full meals per day for all family members round the year • Households monthly income BDT 7,500 or more • Household productive asset value worth BDT 15,000 or more (excluding land, pond and homestead) • Ownership of homestead land 10 decimals or more • Ownership of cultivable land 50 decimals or more (excluding homestead or pond)	If anyone is "NO" go ahead for next questions
Step 3 • Households have married women with in child bearing age (15 to 45 years) • Households have pregnant women (including abandoned or widowed woman) • Households have 0-23 months old children • Households have adolescent girls (15-19 years)	If anyone is 'Yes' go ahead for registration of enrolling as vulnerable Household
Sampling frame was prepared for collecting data from mother-child pair if the households had 0-23 months old children	

Name of all staffs on the project's field site

Field Research Manager: Mohammad Ashraful Islam.

Senior Field Research Officer: Md. Golam Sarwar and Jagadish Chandra Roy.

Field Research Officer: Md. Osman Goni, Md. Saifur Rahman Sarker, Rafiqul Islam Babul, Khaled Saifullah, Md. Morad Hossain, A.B.M. Sharifuzzaman, Md. Nozrul Islam, Md. Abdullah Al Mamun, Md. Abdullah Miah Bablu, Mustafa Mohsin, Shima Sultana and Ms. Taslima Amin.

Field Research Supervisor: Md. Shamim Quamrul Hasan, Shahin Parvez, Md. Belayet Hossain, Pallab Kanti Kundu, Md. Redwan Khan, Md. Hafizur Rahman, Mohammad Noman Siddiki, Md. Faruk Hossain, Syed Murad Ahammad, Shamim Al Mamun, Novel Chandra Das, Md. Taizul Islam, Mohammad Tajkin Ehsan, Prokash Chandra Sarker and Muahammad Waliullah.

Medical Technologist: Md. Munsur Helal, Md. Fazle Rabbi, Al Amin, Marouf Khan, Ashraful Haque, Md. Hafizur Rahman, Md. Jahid Hasan, Mohammad Sajjad Hossain, Kamrul Islam, Md. Rakibul Islam, Md. Mostafijur Rahaman and Prianka Rani.

Field Research Assistant: Arifun Nahar, Maksuda Akter, Mossa Joytsna Akhter, Fahima Khatun, Rojina Akhter, Alaya Akter, Taslima Akter, Rita Begum, Shilpy Akter, Sultana Parveen, Salma Aktar, Saleha Akter, Marium Akter, Raihana Akther, Rashida Akter, Moslama Khatun, Farida Begum, Afsana Akter, Zabaida Akther Jeba, Mst. Nachima Khatun, Most. Marina Khatun, Tanzima Begum, Khadiza Akhter, Taslima Khatun, Israt Jahan Siddiqa, Tanjima Akter, Aklima Akter, Most. Afroza Khatun, Mariam Akhter, Nadira Akter, Majeda Begum, Taslima Khanom, Mst. Nasima Khatun, Sanchita Barua, Bilkis Banu, Rabia Khatun, Afroza Begum Nilu, Nigat Sultana, Shirin Sultana, Mst. Lutfa Begum, Nasrin Sultana, Most. Nadianur Sumi, Md. Seddiqur Rahman, Md. Husain, Prantush Barman, Md. Rejaul Karim, Muhammod Eunus, Azmir Shah, Md. Aminul Islam, Mohammad Ekram Ullah Miah, Md. Asraful Islam, Tapan Kumar Biswas, Bulbul Ahmed, Mazharul Islam, Golam Mostafa, Md. Delwar Hossain, Shishir Louis Piris, Sharif Uddin, Md. Humayun Kabir, Gazi Md. Shawkat Hossain, Imrul Hasan, Md. Aslam Hossain, Ms. Tahera Khatun, Nashrin Akter, Ayesha Begum, Sidratul Muntaha, Forida Easmin, Khadija Akter, Nurjahan Akter, Tamanna Akter, Sadia Islam Nilima, Nowshin Jannat, Medha Roy, Saimun Akter, Sujia Khatun, Mst. Salma khatun, Sushmita Rashid Jhinuk, Mst. Mina Khatun, Mafuza khanam, Sarmin Jahan, Shamima Akhtar, Salma Akter Shanta, Nuran Naher, Jannatul Ferdousi, Rakhi Saha, Jannatul Ferdouse, Sarmin Akter Sathi, Md. Rasel, Mohammad Monir Hossain, Musabbir Ahmed, Md Mahmudul Hassan, Sadia Efat, Borna Thigidi Jhora, Samsun Nahar, Mehenaz Pervin, Umme Soumayia Islam, Md. Abul Kalam Azad, Mohammad Ali Nafi, Saymunnaher, Shahriar Afroj, Tania Ahamed, Nasir Ahmed, Nasrin Akhter, Mst. Nasrin Akhter, Jesmin Aktar, Rina Akter, Mahmuda Akter, Masoda Akter, Sheikh Nargis Akter, Shahinoor

Akter, Parul Akter, Jasmin Akter, Sadia Arefin, Asima Bairagee, Taslima Khnam, Jarjina Begum, Mst. Bulbuli Begum, Rukshana Begum, Bithika Biswas, Md. Emran Choudhury, Sultana Fardushe, Nahid Ferdash, Md. Maynul Haque, Junayed Hossain, Nur-E-Afroza Huda, Mohammad Shahidul Islam, Taslima Akther, Nazrul Islam, A.B.M. Ashiqul Islam, Labani Khanam, Mania Madhu, Popy Marma, Shahnaz Parvin Munni, Madhabi Rani Nath, Shahnaj Parvin, Fatema Shelly, Nusrat Salma Sultana, Humayara Taslim, Shafia Zerin, Shirin Sultana, Sayeada Nurunnahar, Masuma Begum, Jannatul Fardous, Nowshin Monir, Taslima Akhter, Lubna Sultana and Tahmina Begum.
